# What should be tested in patients with suspected mold exposure? Usefulness of serological markers for the diagnosis 

**DOI:** 10.5414/ALX02298E

**Published:** 2022-03-29

**Authors:** Sabine Kespohl, Verena Liebers, Silke Maryska, Ursula Meurer, Claudia Litzenberger, Rolf Merget, Monika Raulf

**Affiliations:** Institute for Prevention and Occupational Medicine of the German Social Accident Insurance, Institute of the Ruhr University Bochum (IPA), Bochum, Germany

**Keywords:** asthma, IgE-mediated sensitization, inflammatory marker, mold exposure, respiratory symptoms

## Abstract

The associations of mold exposure, IgE-mediated sensitization, inflammatory markers, and respiratory symptoms were analyzed in 46 exposed and 23 non-exposed individuals. Both exposure and clinical symptoms were assessed by questionnaire. Specific (s)IgE to mold mixture (mx1) was significantly higher and found more frequently in exposed (41%) than non-exposed individuals (17%), which was not observed for sIgG to mold mix (Gmx6). Notably, exposed asthmatics were more frequently sensitized to molds (55%) compared to exposed non-asthmatics (18%). In addition, the serum concentrations of club cell protein (CC16) were significantly lower in exposed subjects, especially in asthmatics. Positive associations were observed among mold sensitization, asthma, and mold exposure, but not in subjects with predominantly environmental sensitizations without mold sensitization. Thus, sIgE to mx1 but not sIgG to Gmx6 is a useful diagnostic marker to verify mold-associated respiratory symptoms.

## Introduction 

Indoor mold growth has been shown to be a potential health risk, and there is a large number of epidemiological studies on mold-induced health risks and effects, as reviewed in [1, 2] that support and provide evidence linking mold exposure and health risks as reported by the Institute of Medicine (IOM) [3] and the World Health Organization (WHO) [4]. Nevertheless, the question remains how to test and treat patients with respiratory complaints when exposure to mold is suspected or cannot be ruled out as the underlying cause. If patient history points towards mold exposure, an accurate diagnosis has to be established. Relevant issues to be addressed include the cause of the patient’s complaints, and to determine the trigger of the adverse health effects – sensitization, allergy, exposure to irritants or toxic mold components, or an interaction of mold and bacteria. There are few commercial test allergens available to perform skin tests for suspected mold allergy, and even less mold extracts for provocation tests [5, 6]. To detect toxic/irritant effects, it is necessary to know the responsible mold species and to be able to quantify the exposure, which is usually not possible. Furthermore, it is not known if a retrospectively collected air or dust sample can provide information on exposure, or whether there are immunological parameters that can indicate mold and/or bacterial exposure. Other open questions include how to verify a patient’s suspicion of exposure to molds, to determine whether mold-specific IgG is useful, to ascertain if the exposure occurred at the workplace, and whether it is at all necessary to distinguish between occupational exposure and exposure at home. Regardless, the specific causes of the exposed patient’s complaints should be identified and where possible, the disease as well. Accordingly, the German guideline for clinical diagnosis of indoor mold exposure [7] recommends objectifying mold-/dampness-induced health problems, such as asthma, allergic rhinitis, hypersensitivity pneumonitis, and respiratory infections. The following aspects should be inquired about in the medical history: history of exposure in the living, working, and recreational areas; allergy and infection history, including predisposing factors; history of irritative-toxic and odor effects and disturbances in well-being. 

Since the composition of bioaerosols, including indoor dust, is very complex, it becomes extremely difficult to accurately evaluate exposure to molds and/or microbial components [8]. On the basis of the current state of knowledge, risk analysis and assessment of indoor mold exposure can only be carried out as semi-quantitative risk assessment. Concrete numerous data for mold species or mold components by mean of dose-response relation are not available [9]. Therefore, a quantitative mold exposure measurement is not recommended as a general strategy, although exceptions should be made in individual cases of concrete health problems and existing suspicion of causal mold exposure. It is rather recommended to consider indoor mold growth as a potential health risk, even if a quantitative and causal relationship cannot be established between the occurrence of individual species and health conditions [7]. In this respect, questionnaire-based data on dampness, moisture, and microbial exposure can be a useful and cost-effective alternative. Several ECRHS studies have shown that visible indoor mold or dampness assessed by questionnaires were associated with increased prevalence of asthma symptoms, diagnosed asthma, and bronchial hyper-responsiveness [1, 10, 11]. In particular, indoor mold exposure in asthmatics has a severe impact on respiratory symptoms, i.e., exacerbation and aggravation [12]. Although questionnaire-based data on indoor mold exposure are not as sensitive as expert-collected data [13], they can serve as adequate indicators of the characteristics of the indoor environment [14]. 

The importance of mold sensitization in patients with respiratory symptoms was emphasized in a recent study of 3,358 patients who were tested over a 20-year period for various (indoor and outdoor) molds using skin prick tests [15]. While 19.2% of the patients were sensitized to at least one of the fungal species of *Alternaria*, *Aspergillus,* or *Cladosporium* during 1998 – 2007, this rate increased to 22.5% during 2008 – 2017. Looking at the mold species individually, there is a moderate increase in the prevalence of sensitization in the second decade against outdoor molds *Alternaria alternata* by 36% (from 8.6 to 11.7%) and *Cladosporium herbarum* by 22% (from 8.2 to 10%). But for indoor mold species, the increase in sensitization rates is much higher; for *Aspergillus fumigatus* by 42% (from 7.3 to 10.2%) and for *Penicillium chrysogenum* even by 127% (from 12.6 to 28.6%). Typical indoor mold species like *Aspergillus fumigatus* and *Penicillium chrysogenum* should be considered in routine testing accordingly, if a fungal allergy is suspected. Since skin prick test for mold allergens are becoming increasingly rare, serological tests are the only alternative. In a previous study, it was shown that IgE testing with mx1 mold mixture is a valid tool for screening for potential mold sensitization [5]. 

Next to IgE-mediated pathomechanisms, mold exposure can also induce inflammatory health effects. Useful markers to monitor pro-inflammatory and anti-inflammatory effects as early sings of lung damage after dust/microbial exposure were investigated recently [16, 17, 18, 19]. Human bronchial epithelial cell line exposed to spores and hyphal fragment from different fungal cultures showed that *Aspergillus fumigatus* and *Pencillium chrysogenum* hyphae in particular induce the expression and release of interleukin (IL) 6 and chemokine IL-8 [16]. In another recent study, an association was found between serum amyloid A (SAA) and C-reactive protein (CRP) levels after occupational exposures to various fungi and bacterial genera in greenhouse workers [17]. Both the composition of the fungal and bacterial organisms and the level of exposure affected the serological levels of SAA and CRP in exposed workers. Another promising serological biomarker for the early indication of lung damage is the anti-inflammatory club cell protein 16 (CC16) [20], which has been analyzed in several studies of occupationally exposed workers. Club cells are secretory cells in the bronchioles of the lung that are involved in the non-specific defense against harmful substances. In the process, club cells secrete, among others, a 16 kDa protein (CC16) first into the alveolar lavage fluid, from where it reaches the serum through a constant diffusion rate that depends on the permeability of the bronchial epithelium. If the bronchial epithelium and thus the permeability is damaged by mechanical or chemical/toxic factors, or if there is an increased death of club cells, this can be measured at an early stage by the serological concentration of the CC16 protein. It was shown, that the concentration of CC16 in serum was reduced in a dose-dependent manner by tobacco smoking and was significantly lower in asthmatics compared to healthy subjects [21]. Therefore, the intention of the present study was to investigate which serological parameters could be useful in order to evaluate and classify mold-induced respiratory symptoms in exposed patients. For this purpose, mold/dampness exposure and corresponding respiratory symptoms were recorded by questionnaire and the serological parameters mentioned above were determined. 

## Materials and methods 

### Study group 

In total, 69 subjects were included. Exposed subjects with suspected mold- and/or dampness-associated respiratory symptoms from our clinical center at IPA or staff (n = 55), as well as non-exposed healthy subjects without known mold exposure and without known mold-associated respiratory symptoms (n = 14) from IPA staff, or students were asked to participate. The classification into mold/dampness exposed and non-exposed was not based on recruitment but on exposure data provided by a questionnaire that potential participants were asked to complete. Finally, the exposed group consisted of 46 participants and the non-exposed group of 23. All participants provided written approval for participation, and the study itself was approved by the ethics committee of the Ruhr University Bochum (register no. 4231-12) and was conducted in accordance with the Helsinki Declaration. 

### Questionnaire-based respiratory symptoms induced by mold/dampness 

Mold/dampness-induced symptoms were documented using a questionnaire based on the following questions: 

Do you suffer from the following complaints?

- Sneezing, itching in the nose, rhinorrhea (runny nose), nasal congestion
- Redness, itching of the eyes, tears
- Whistling or buzzing noise in the chest
- Cough (productive, with sputum)
- Dry cough
- Asthma, shortness of breath, heavy breathing
- Shortness of breath after strenuous activities
- Shortness of breath at rest
- Chest tightness
- Skin rash or eczema
- Repeated fever attacks, flu-like symptoms
- Melalgia (joint pain)
- Other

Affirmative responses to questions 1 and/or 3 – 8 were categorized as respiratory symptoms. Asthma was diagnosed with a positive answer to symptom 6, as well as by answering “yes” to the question if the test person was ever diagnosed with asthma by a doctor, and if the person was currently taking any medication for respiratory symptoms or allergies. By using a strict definition of asthma , subjects with undiagnosed asthma might be missed in this group. However, this strict classification makes the link between asthma and mold exposure more stringent. 

### Questionnaire-based determination of mold exposure or dampness in homes 

To establish mold exposure, visible mold infestation and dampness were required at both the workplace and in private living areas, as determined with the following questions:

- Was/is there visible mold infestation at your workplace/living area?

- Answering with “yes” resulted in categorization into the exposed group; subjects who answered “no” were grouped in the non-exposed group

- If yes, then the size of infestation was considered – whether the area is/was smaller than 21 × 29.7 cm (corresponding to the national standard DIN A4) or larger that DIN A4, and whether it was present at the time of investigation or had occurred in the past.

- Answering larger DIN A4 meant “extensive exposure”;
  - Answering currently and smaller than DIN A4 or not specified meant “currently exposed”;
  - Answering in the past and smaller than DIN A4 or not specified indicated “low exposed”;

- The final question asked whether the following are/were present in the workplace/living area: humidifier, indoor/table fountain, air conditioning, aquarium

- Answering with “yes” in the non-exposed group suggested non-exposed and dampness
  - Answering with “no” in the non-exposed group signified non-exposed and no dampness

In addition, there were questions on potential exposures at the workplace in order to investigate hidden sources of mold exposure. Subjects with professions in the following industries were automatically categorized as “extensively exposed”: Agriculture with or without animals, garbage or sewage plants, and food processing work using molds, e.g., cheese or sausage production. 

### Serological parameters 

Inflammatory parameters were measured in all serum samples using human CC16, human SAA, and IL-6. CC16 was determined with a human club cell protein ELISA (BioVendor-Laboratorni dimicina a.s., Czech Republic), and SAA was measured with the human SAA ELISA kit (Invitrogen, CA, USA) according to manufacturers’ instructions. IL-6 was measured with the human IL-6 DuoSet ELISA (Bio-Techne, R&D Systems, Minneapolis, MN, USA), and values above the detection limit of 4.7 pg/mL were classified as positive. 

Total IgE (tIgE) and specific IgE (sIgE) concentration against a mixture of ubiquitous environmental allergens (sx1; including *Dermatophagoides pteronyssinus*, cat, dog, timothy grass, rye, *Cladosporium herbarum*, birch, mugwort) and mold allergens (mx1, including *Penicillium chrysogenum, Cladosporium herbarum, Aspergillus fumigatus, Alternaria alternata)* were measured using ImmunoCAPs (ThermoScientific, Uppsala, Sweden). Atopic status was defined as sIgE to sx1 values ≥ 0.35 kU_A_/L. Mold sensitization was defined as sIgE values to mx1 ≥ 0.35 kU_A_/L. Serum samples with mx1 ≥ 0.35 kU_A_/L were additionally tested for relevant indoor mold species, *Aspergillus fumigatus* (Asp f) and *Penicillium chrysogenum* (Pen ch) using ImmunoCAPs, as well as for the typical outdoor mold species, *Alternaria alternata* (Alt a) and *Cladosporium herbarum* (Cla h). Specific IgG was measured as potential screening tool for mold exposure. The only commercially available mold mixture for measuring sIgG was Gmx6 by ImmunoCAP, including *Penicillium chrysogenum*, *Cladosporium herbarum*, *Mucor racemosus*, and *Alternaria alternata *but not* Aspergillus fumigatus*. sIgG values were considered as increased if values were ≥ 32 mg_A_/L corresponding to 97.5% percentile of a healthy non exposed reference group as previously reported [22]. 

### Statistical analysis 

A total of 529 serological values were analyzed, and median with range and/or cut-off values were given. Comparison of values in different exposure groups, as well as among sub-groups were analyzed using GraphPad Prism 9.3.1 Calculation of significant differences among sIgE values was done with Mann-Whitney test (two-group comparison) or Kruskal-Wallis test (multiple group comparison) or with χ^2^-test for frequency rate by GraphPad Prism 9.3.1. 

## Results 

### Mold and dampness exposure assessment by questionnaire 

**Figure 1:**
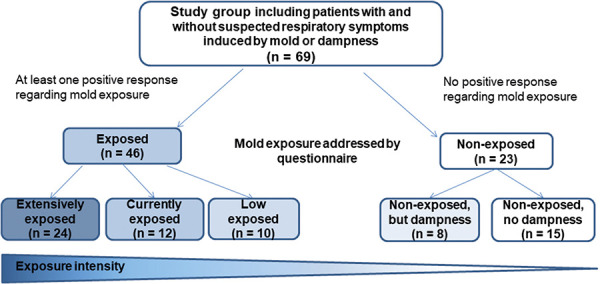
Diagram of exposure group classification based on questionnaire data about mold and dampness.

Indoor mold exposure and dampness were documented using a self-reported questionnaire, resulting in the categorization of different groups. The exposed group consisted of 46 subjects, with at least one reported mold exposure (home and/or workplace); the non-exposed group consisted of 23 subjects without any reported mold exposure (Figure 1). 

**The exposed group (n** **=** **46)** was subdivided into:

- **extensively exposed** (n = 24): with visible mold infestation of a size greater than DIN A4 in homes and/or workplaces
- **currently exposed** (n = 12): with visible mold infestation smaller than the size of DIN A4 in homes and/or workplaces
- **low exposed** (n = 10): not currently exposed, and mold exposure was smaller than DIN A4 dimensions in homes and/or workplaces

**The non-exposed** group **(n =** **23)** was subdivided into:

- **non-exposed, but dampness** (n = 8): with no visible mold exposure, but dampness at home and/or at workplace
- **non-exposed, no dampness** (n = 15): with no visible mold exposure, and dampness neither at home nor at the workplace

### Serological data on inflammation and sensitization in exposed vs. non-exposed subjects 

Characteristics and serological data of both groups are summarized in Table 1. Age (median age 48 vs. 45 years), gender (48% females in both groups), and smoking habits (13% in both groups) were comparable in both the exposed and non-exposed subjects. In the former, questionnaire-based respiratory symptoms (94 vs. 52%) and asthma (63 vs. 22%) were reported more frequently in the exposed compared to the non-exposed group. 

CC16 concentrations measured in sera of exposed subjects were 7.7 ng/mL and thus significantly lower than in the non-exposed subjects (10.2 ng/mL). SAA concentrations were not different between both groups. IL-6 concentrations were analyzed in the sera of both the exposed and non-exposed subjects and found to be below the detection limit in 74% and 70%, respectively. The number of subjects with elevated sIgG concentration to Gmx6 as a potential marker of mold exposure was comparable in both groups or even lower in the exposed group than in the non-exposed group. However, the two groups differed significantly with regard to IgE levels. In the exposed group, IgE values were significantly higher for tIgE and sIgE to mx1. Furthermore, mold sensitization (sIgE to mx1 ≥ 0.35 kU_A_/L) occurred more frequently in the exposed subjects (41%) than in non-exposed subjects (17%). However, the groups did not differ significantly with regard to IgE reactivity to sx1. 

**Figure 2:**
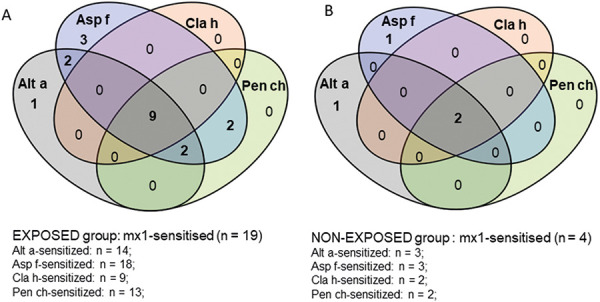
A, B: Venn diagram of individual mold sensitization (≥ 0.35 kU_A_/L) to *Alternaria alternata* (Alt a), *Aspergillus fumigatus* (Asp f), *Cladosporium herbarum* (Cla h), and *Penicillium chrysogenum* (Pen ch). All subjects with mold sensitization (sIgE to mx1 ≥ 0.35 kU_A_/L) of the exposed group n = 19 (A) and the non-exposed group n = 4 (B) were tested.

Sera from subjects who had a positive sIgE response to mx1 were re-tested for sIgE to the individual mold species which were included into the mx1 mixture. These were the typical indoor molds *Aspergillus fumigatus* and *Penicillium chrysogenum* as well as the typical outdoor molds *Alternaria alternata* and *Cladosporium herbarum.* Sensitizations to individual molds were investigated in 19 of 46 exposed and in 4 of 23 non-exposed participants. The results are shown in Figure 2. In both groups, ~ 50% had sIgE to all four tested mold species. Monosensitization was only observed for *Aspergillus fumigatus* and *Alternaria alternata* in both exposure groups, but not to *Cladosporium herbarum* or *Penicillium chrysogenum*. Overall, the most frequent sensitization was observed for *Aspergillus fumigatus* in the exposed group with 18 of 19 mx1-sensitized subjects (95%) and 3 out of 4 (75%) in the non-exposed group. This was followed by sensitization to *Alternaria alternata* with 14 out of 19 (74%) in the exposed vs. 3 out of 4 (75%) in the non-exposed subjects. Sensitization to *Penicillium chrysogenum* was more pronounced in the exposed group with 68% compared to 50% in the non-exposed group. For *Cladosporium herbarum,* sensitization of ~ 50% was found only together with all other mold species tested. 

**Figure 3:**
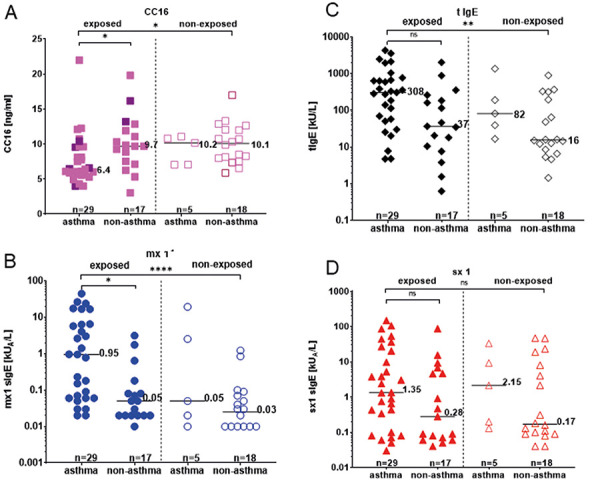
Serological concentration (from left to right) of club cell protein (CC16) (A), sIgE to mx1 (B), tIgE (C), and sIgE to sx1 (D) in exposed and non-exposed subjects stratified by asthma. Significant differences were labeled with *p ≤ 0.05, **p ≤ 0.01, and ****p ≤ 0.0001.

Significant associations between mold exposure and asthma were analyzed in detail by subdividing the exposed and non-exposed group into asthmatics and non-asthmatics with regard to CC16, tIgE as well as mx1 and sx1 sIgE (Figure 3). In the group of exposed subjects, those with asthma had significantly lower CC16 levels (Figure 3A). In contrast, no significant difference could be detected between asthmatics and non-asthmatics in the group of non-exposed subjects. It should be noted, however, there were only five subjects in the non-exposed group suffered from asthma. Even though there was no significant difference between asthmatics with and without exposure, the median values were indeed different (6.4 ng/mL in exposed asthmatics vs. 10.2 ng/mL in non-exposed asthmatics). With respect to mold sensitization (sIgE to mx1 ≥ 0.35 kU_A_/L), more frequent sensitization and higher IgE values were detected in the exposed asthmatics than in the exposed non-asthmatics (median values of 0.95 kU_A_/L vs. 0.05 kU_A_/L) (Figure 3B). This effect was particularly significant for exposed asthmatics. In contrast, no difference was detected between asthmatics and non-asthmatic in the non-exposed group. tIgE concentrations (Figure 3C) in asthmatics in the exposed group were higher than in the non-exposed group, but the effect was only significant when exposed asthmatics were compared to non-exposed non-asthmatics. sIgE values to ubiquitous allergens (measured by sx1) were also higher, albeit not significant, in asthmatics independent of exposure (Figure 3D). 

### Respiratory symptoms, mold exposure, and serological values in relation to sensitization 

Of the 69 subjects studied, a total of 43 were sensitized to at least 1 allergen mixture (environmental allergens (sx1) and/or mold mixture (mx1)), and in 26 subjects no sensitization to any allergen source was measured neither to mx1 nor to sx1 < 0.35 kU_A_/L (Table 2). Of the 43 sensitized subjects, 23 were classified as predominantly sensitized to molds as shown by mx1 sIgE ≥ 0.35 kU_A_/L; 20 were predominantly sensitized to environmental allergens like pollen, mites, animal dander, etc. based on sx1 ≥ 0.35 kU_A_/L and mx1 ≤ 0.35 kU_A_/L. Respiratory symptoms (58%) and asthma (31%) were significantly less frequent in non-sensitized subjects compared to sensitized subjects (predominantly sensitized to environmental allergens and/or predominantly mold sensitized). However, in asthma patients, a significantly higher rate was seen in predominantly mold sensitized subjects compared to patients predominantly sensitized to environmental allergens. 78% of the mold-sensitized subjects reported that they suffered from asthma, whereas only 40% of subjects predominantly sensitized to environmental allergens and 31% of the non-sensitized group reported to have asthma. Reported mold exposure was also more common at 83% than in individuals predominantly sensitized or non-sensitized to environmental allergens, of whom around 60% reported mold exposure. However, this difference was not significant, nor were sIgE values to sx1, tIgE, or serological SAA and IL-6 concentration. However, CC16 values were lower in predominantly mold-sensitized individuals than in non-sensitized subjects. 

### Serological parameters and respiratory symptoms in different exposure sub-groups 

In order to better assess the influence of mold exposure on individual parameters, we assigned the subjects to sub-groups according to their exposure levels (Table 3A, Table 3B). Among patients in the exposed sub-groups, more IgE and IgG parameters were positive (according to the respective criteria) in patients categorized in the extensively and low exposed sub-groups compared to the currently exposed sub-group (Table 3A). Among the exposed sub-groups, SAA concentrations (median) were highest in the low-exposed sub-group; whereas, the highest levels of CC16 were found in the currently exposed sub-group. The rather weak influence of the current, small-scale exposure within the exposed sub-groups was striking. The further subdivision of the non-exposed group showed the highest values of CC16 in the non-exposed no dampness sub-group. However, all the observed trends were not significantly different due to small group size. 

In contrast, reported respiratory symptoms in exposed and non-exposed sub-groups were associated with exposure intensity (Table 3B). With respect to the individual symptoms, dry cough, asthma, and shortness of breath (after activity) are the most frequently reported complaints, which increase in frequency in the same way than questionnaire-based exposure intensity was reported. Upper respiratory symptoms, such as rhino-conjunctivitis (nose, eyes) or skin symptoms tended to be more independent of exposure. Chest tightness, repeated fever, and flu-like symptoms were more frequently observed in patients of the exposed sub-groups, especially flu-like symptoms in the currently exposed, which could be an indication of hypersensitivity pneumonitis (type III and IV allergy) possibly induced by other fungal or bacterial species and should be further investigated. 

Considering that occupational exposures are usually more intense and longer than domestic exposures, this difference was investigated focusing on respiratory symptoms and serological values (Table 4). 74% of occupationally exposed subjects reported extensive exposure compared to 46% reporting extensive exposure in homes. Furthermore, respiratory symptoms and asthma were reported significantly more frequently in the occupationally exposed group compared to those exposed at home (respiratory symptoms 100 vs. 77%; asthma 87 vs. 46%). However, this was not reflected in sIgE sensitization to mold (mx1), which was similar in both groups at 39%. However, the median IgE value to *A. fumigatus* was significantly higher in the occupationally exposed group (3.23 vs. 0.69 kU_A_/L). Other sIgE values for *P. chrysogenum* and relevant outdoor mold species, *A. alternata* and *C. herbarum,* were comparable in both groups. Finally, the inflammatory marker CC16 was lower in the occupationally exposed subjects (median value: 6.08 vs. 8.86 ng/mL), which corresponded to the higher proportion of asthmatics in the occupational group. 

## Discussion 

The intention of the current study was to investigate associations of mold exposure, IgE sensitization, inflammatory markers, and respiratory symptoms. The results were used to determine serological parameters that might be useful for diagnosis. 

### Mold exposure assessment 

The questionnaire-based estimation of mold exposure is difficult and may include bias factors, especially when patients suspect that mold is the trigger of their health problems. Various studies [23, 24, 25] showed that a questionnaire-based exposure assessment by tenants or parents of children with respiratory symptoms resulted in more frequent reporting of dampness and mold compared to the results from trained inspectors or viable fungi measurements, especially when the exposure to dampness/mold was low. This bias could imply that mold exposure causes respiratory symptoms, but the studies suggest that their presence may lead to an over-reported mold exposure. On the other hand, studies also showed that visible indoor mold identification and moisture sources corresponded with elevated levels of indoor molds [14, 25, 26]. Thus, in order to objectify mold exposure, questionnaire-based mold exposures were evaluated for plausibility on the basis of the individual medical history. For this purpose, either mold-associated workplaces or visible mold infestation in inhabited indoor spaces of the patients were considered as criteria for mold exposure. sIgG against the mold mix (Gmx6) was measured as a serological confirmation or possible biomarker of exposure. However, elevated IgG values against the mold mix Gmx6 were measured in 7 of 69 subjects, of which 5 belonged to the exposed group and 3 to the non-exposed group. It can therefore be concluded that the determination of sIgG against Gmx6 is not an effective marker for mold exposure, and we cannot give a general recommendation for the determination of Gmx6 sIgG in cases of suspected mold exposure. 

### Mold exposure and the serological parameters total IgE, sx1, mx1 

Of the exposed subjects, 93% were recruited from the clinic, while the non-exposed subjects were mainly staff or students and only 43% were recruited from the clinic. This is a clear weakness of the study, as a symptom-based pre-selection is made among exposed subjects with regard to the frequency of respiratory symptoms and asthma. Accordingly, serological parameters showed more frequently mold sensitization (mx1) and elevated tIgE values with significantly higher IgE values in the exposed group compared to the non-exposed group (Table 1). Stratifying these effects for asthma, a significant difference was seen only for mold sensitization (Figure 3C). These results are consistent with previous research by Jaakkola et al. [27], who showed that asthma risk in adults increased significantly with atopy in a dose-dependent pattern, measured as increasing values of tIgE and of sIgE to sx1. Furthermore, Jaakkola et al. [27] calculated an increased OR of asthma in subjects with sensitization to mite and mold as well, and for any mold sensitization adjusted OR of asthma was 2.01 (1.11 – 3.67). In particular, high sIgE- oncentration to *Aspergillus fumigatus* increased OR by a factor of 2.7 (1.38 – 5.4). Overall, the detection of mold sensitization is an important tool to investigate suspected mold-associated respiratory symptoms. In one of our previous studies [5], measurement of the mold mixture mx1 containing allergens from *Aspergillus fumigatus, Penicillium chrysogenum, Cladosporium herbarum,* and *Alternaria alternata* revealed that mx1 identified all sensitizations to individual mold components. Therefore, mx1 is an appropriate screening tool to investigate suspected mold sensitization and can be recommended as serological parameter. Determination of tIgE also showed increased values in the exposed patient groups in association with mold exposure, but this was not statistically significant. Even if a selective effect has to be considered, other studies have also shown an association between mold exposure, mold sensitization, atopy, increased concentration of tIgE, and asthma. On the one hand, mold exposure triggers asthma onset in children and young adults [1, 2, 27, 28, 29, 30], on the other hand, severe asthma symptoms are often associated with mold sensitization [31, 32]. But also a decline in lung function without asthma has been measured as a result of indoor mold exposure [33]. Next to IgE-mediated respiratory symptoms, which are probably based on mold sensitization, further immunological mechanisms were suspected to be involved in respiratory health conditions. This is supported by the fact that mold-associated symptoms occur more frequently than IgE-mediated mold sensitization. Therefore, further characterization of in vitro cellular activation was performed in whole blood assay in part of the study group and has been published by Liebers et al. [34]. 

### Mold exposure and respiratory symptoms 

In order to more thoroughly investigate mold exposure, the subjects were divided into groups with different exposure intensities (Table 3B). It became clear that the extensively exposed subjects reported more frequently respiratory symptoms and asthma, with the frequency decreasing in the same manner than mold exposure. In the non-exposed group without dampness, only 27% reported respiratory symptoms, whereas in the extensively exposed sub-group, up to 75% reported symptoms. In particular, the rate of asthmatics was reduced in the same way – there were one-third fewer asthmatics in the currently-exposed and low-exposed groups compared with the extensively-exposed group. A similar effect of building dampness and mold exposure was described in a meta-analysis [35], which reported a 30 – 50% increase in various respiratory and asthma-related health outcomes with statistically significant association to mold exposure in nearly all cases. Even though the results of the current study might be supposed to reflect the pre-selection of exposed subjects, similar effects have also been shown in other studies [10, 36]. 

### Occupational exposure 

In occupationally mold-exposed patients, the rate of mold sensitization was comparable to that of non-occupational but home-exposed patients. However, sensitization to *Aspergillus fumigatus* in particular was associated with occupational exposure, whereas sensitization to *Alternaria alternata* occurred predominantly in mold-sensitized patients without occupational exposure. This was confirmed by significantly increased sIgE values to *Aspergillus fumigatus* (median values: 3.23 kU_A_/L in occupationally exposed vs. 0.69 kU_A_/L in non-occupationally exposed individuals). Asthma occurred more frequently in occupationally exposed subjects and suggests an association with *Aspergillus fumigatus* sensitization as well. Results of a case-control study [29] support the prominent role of *Aspergillus fumigatus* in asthmatics. In this study, it was shown that 50% of *Aspergillus fumigatus*-sensitized asthmatics had severe asthma vs. 15.6% of non-sensitized asthmatics with an OR of 5.4 (1.00 – 29.06; p = 0.059). Occupational mold exposure is considered to be extensive and prolonged, and exposed subjects reported respiratory symptoms and asthma more frequently. Indeed, the increased risk of adult-onset asthma was previously described in relation to mold exposure at workplaces, but not in homes [37, 38]. The relative risk of mold-induced asthma could be stratified according to the mold species *Aspergillus fumigatus* and *Cladosporium herbarum* [27]. The risk for the onset of new asthma increased proportionally with sIgE level to *Aspergillus fumigatus* [27]. The association between sensitization to *Aspergillus fumigatus* and asthma should be considered in patients with respiratory symptoms and occupational mold exposure. 

### Mold exposure and inflammatory markers 

Systemic inflammatory biomarkers have been proposed as diagnostic tools for different diseases, e.g., chronic obstructive pulmonary disease. In the current study, we measured SAA as an acute-phase protein, IL-6 as an inflammatory marker, and secreted CC16 as a pneumoprotein that is synthesized predominantly in the lungs and found in serum. Both biomarkers, SAA and IL-6, exhibited comparable median values and ranges in exposed and non-exposed groups and were therefore independent of mold exposure in our study group. However, this was not true for the median values of CC16, which were significantly lower in the exposed group compared to the non-exposed group. It has been previously reported, that CC16 expression decreased with lung injury and is thus considered a marker of bronchial cell dysfunction [39]. Increased permeability of the lung epithelial barrier can be caused by toxic/microbial exposure, but in addition there are other confounding factors, such as smoking and asthma, or age, gender, or exercise, which has to be considered [19, 21, 40]. Our current study groups were comparable in age and gender. Even after the exclusion of smokers, there was a significant decrease in CC16 concentration in the exposed group. In contrast to smoking, which was equally prevalent in both the exposed and non-exposed groups at 13%, respiratory symptoms – specifically rhinitis, cough, shortness of breath and asthma – were very common in more than 90% of the exposed but only in ~ 50% of the subjects in the non-exposed group. When subjects with reported asthma were excluded from both exposure groups, serological CC16 levels showed no further differences in median values (exposed non-asthmatics: 9.7 ng/mL vs. non-exposed non-asthmatics: 10.1 ng/mL). The decrease in CC16 concentration with increasing exposure to molds observed in our study is most likely due to the higher proportion of asthmatics in the exposed sub-groups and not to the intensity of exposure. Thus, CC16 is not a useful marker for mold exposure but, in our group, for asthma. This is consistent with an earlier study [40] showing that serological CC16 concentrations were significantly decreased in asthmatics (atopic and non-atopic), especially for longer duration of asthma (≥ 10 years). 

**Figure 4:**
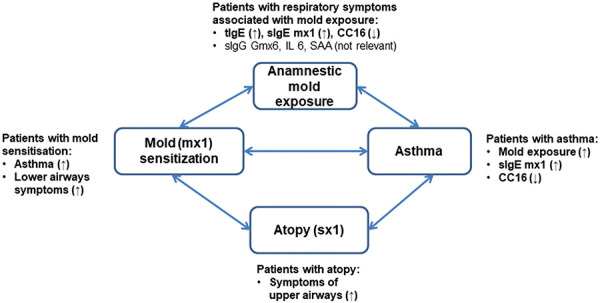
Association of mold exposure, asthma, atopy, and mold sensitization. (↑): more frequently and/or higher concentration; (↓) less frequently and/or lower concentration.

In conclusion, the following associations between mold exposure, sensitization, and asthma can be derived from our study (Figure 4). Patients who complained of respiratory symptoms related to mold exposure were more likely to report asthma, more likely to be mold sensitized, and more likely to have significantly lower CC16 levels. In mold-sensitized patients, lower respiratory tract symptoms were particularly emphasized. Furthermore, 82% of mold-sensitized patients were poly-sensitized and atopic. Atopic patients in turn had increased upper respiratory tract problems, which could subsequently lead to asthma due to the allergic march. Asthmatics were significantly more likely to have elevated levels of tIgE and sIgE-mediated sensitization to molds, but significantly lower CC16 levels. Furthermore, patients with asthma were more frequently exposed to molds. 

Based on this association, it is recommended that the clinician first perform serological sIgE measurement for mold mix (mx1). If mold-induced allergic asthma is suspected, further clarification of IgE-mediated sensitization should be performed, starting with sIgE or skin prick testing for individual mold allergens and ending with bronchial challenge test. In the case of occupational mold exposure, additional testing of sIgE to *Aspergillus fumigatus* could be recommended in subjects with a positive sIgE response to mx1. Respiratory symptoms of upper airways or skin without reported mold exposure might be associated with sensitization to environmental allergens. Specific IgG to Gmx6, as well as inflammatory markers IL-6 and SAA were not associated with mold exposure and are therefore not useful to include into the diagnostic test panel. 

## Acknowledgment 

We would like to thank the medical-technical colleagues from the clinical center and department of allergology/immunology for their valuable work, the scientific colleagues for their constructive comments, and Dr. Rosemarie Marchan for improving the language. 

## Funding 

The study was supported by the DGUV (German Social Accident Insurance, Sankt Augustin, Germany, IPA-project 145-Bioaerosole). 

## Conflict of interest 

The authors declare no conflict of interest. 

**Table 1:** Characteristics and serological data of the study group in relation to mold exposure.

	Exposed group	Non-exposed group
Number of subjects: n	46	23
Age in years: median (range)	48 (23 – 79)	45 (22 – 63)
Male: n (%)	24 (52%)	12 (52%)
Female: n (%)	22 (48%)	11 (48%)
Smoker: n (%)	6 (13%)	3 (13%)
Respiratory symptoms: n (%)	43**** (94%)	12 (52%)
Asthma: n (%)	29** (63%)	5 (22%)
CC16 (ng/mL): median (range)	7.7* (3.0 – 22.0)	10.2 (5.6 – 17.0)
SAA (ng/mL): median (range)	17,074 (1,880 – 578,521)	16,035 (1,402 – 527,430)
IL 6 (pg/mL): median (range)	4.7 (4.7 – 73.8)	4.7 (4.7 – 1,077.8)
IL 6 > 4.7 (pg/mL): n (%)	12 (26%)	7 (30%)
Gmx6 IgG (mgA/L): median (range)	10.07 (2.11 – 133.8)	10.18 (1.96 – 67.69)
Gmx6 IgG ≥ 32 (mgA/L): n (%)	5 (11%)	3 (13%)
tIgE (kU/L): median (range)	172.7* (0.63 – 4,302)	16.7 (1.45 – 1359)
tIgE ≥ 150 (kU/L): n (%)	24 (52%)	7 (30%)
sx1 IgE (kUA/L): median (range)	0.91 (0.03 – 150.0)	0.20 (0.04 – 47.05)
sx1 IgE ≥ 0.35 (kUA/L): n (%)	29 (63%)	10 (43%)
mx1 IgE (kUA/L): median (range)	0.12*** (0.01 – 44.56)	0.03 (0.00 – 19.22)
mx1 IgE ≥ 0.35 (kUA/L): n (%)	19 (41%)*	4 (17%)

Significant differences are bold and labelled with *p ≤ 0.05, **p ≤ 0.01, ***p ≤ 0.001, and ****p ≤ 0.0001.

**Table 2:** Predominantly sensitized to mold allergens vs. environmental allergens.

	Predominantly sensitized to mold allergens	Predominantly sensitized to environmental allergens	Non-sensitized
Number: n	23	20	26
Respiratory symptoms: n (%)	23 (100%)	17 (85%)	15*** (58%)
Asthma: n (%)	18 (78%)	8* (40%)	8*** (31%)
Exposed: n (%)	19 (83%)	12 (60%)	15 (58%)
sx1 IgE (kU_A_/L): median (range)	3.78 (0.04 – 150.0)	6.04 (0.44 – 87.99)	0.08** (0.03 – 0.31)
tIgE (kU/L): median (range)	592.9 (8.6 – 4,302.0)	128.2 (4.8 – 897.7)	14.87* (0.63 – 283.4)
CC16 (ng/mL): median (range)	7.13 (3.04 – 21.96)	9.79 (5.75 – 16.98)	9.60 (4.00 – 12.87)
SAA (ng/mL): median (range)	19,331 (1,402 – 340,643)	15,525 (1,519 – 527,430)	15,488 (1,880 – 578,522)
IL 6 > 4.7 (pg/mL): n (%)	8 (35%)	8 (40%)	6 (23%)

Significance calculated to predominantly mold sensitized (sIgE mx1 ≥ 0.35 kU_A_/L); significant differences are bold and labeled with *p ≤ 0.05, **p ≤ 0.01, and ***p ≤ 0.001.

**Table 3A:** Serological values in exposed and non-exposed sub-groups.

	Exposed sub-groups			Non-exposed sub-groups	
	Extensively exposed	Currently exposed	Low exposed	Non-exposed, but dampness	Non-exposed, no dampness
Number: n	24	12	10	8	15
Total IgE ≥150 (kU/L): n (%)	13 (54%)	5 (42%)	6 (60%)	3 (38%)	4 (27%)
sx1 IgE ≥ 0.35 (kU_A_/L): n (%)	17 (71%)	5 (42%)	7 (70%)	6 (75%)	4 (27%)
mx1 IgE ≥ 0.35 (kU_A_/L): n (%)	9 (38%)	3 (25%)	7 (70%)	1 (13%)	3 (20%)
Gmx6 IgG ≥ 32 (mg_A_/L): n (%)	3 (13%)	0 (0%)	2 (20%)	1 (13%)	2 (13%)
IL 6 > 4.7 (pg/mL): n (%)	6 (25%)	4 (33%)	2 (20%)	2 (25%)	5 (33%)
CC16 (ng/mL): median	7.34	8.52	7.65	8.13	10.71
SAA (ng/mL): median	18,943	13,503	30,565	32,090	11,527

**Table 3B:** Reported respiratory symptoms in exposed and non-exposed sub-groups.

	Exposed sub-groups			Non-exposed sub-groups	
	Extensively exposed	Currently exposed	Low exposed	Non-exposed, but dampness	Non-exposed, no dampness
Number: n	24	12	10	8	15
Nose: sneezing, itching^R^: n (%)	17 (71%)	8 (67%)	3 (30%)	5 (63%)	4 (27%)
Eyes: tears, itching: n (%)	12 (50%)	7 (58%)	2 (20%)	5 (63%)	3 (20%)
Chest: whistling, buzzing noise^R^: n (%)	9 (38%)	2 (17%)	4 (40%)	1 (13%)	1 (7%)
Cough (productive)^R^: n (%)	9 (38%)	5 (42%)	6 (60%)	3 (38%)	3 (20%)
Cough (chesty)^R^: n (%)	**15 (63%)**	**6 (50%)**	**4 (40%)**	**3 (38%)**	**2 (13%)**
Asthma^1R^: n (%)	**18 (75%)**	**6 (50%)**	**5 (50%)**	**2 (25%)**	**3 (20%)**
Shortness of breath (after activity)^R^: n (%)	**17 (71%)**	**6 (50%)**	**7 (70%)**	**3 (38%)**	**3 (20%)**
Shortness of breath (at rest)^R^: n (%)	4 (17%)	0 (0%)	2 (20%)	1 (13%)	1 (7%)
Chest tightness: n (%)	10 (42%)	1 (8%)	3 (30%)	0 (0%)	1 (7%)
Skin rash (eczema): n (%)	10 (42%)	4 (33%)	1 (10%)	3 (38%)	3 (20%)
Repeated fever, flu-like symptoms: n (%)	6 (25%)	**5 (42%)**	1 (10%)	1 (13%)	3 (20%)

^1^Physician-diagnosed, with medication; ^R^summarized as respiratory symptoms.

**Table 4:** Occupational vs. indoor (home) mold-exposed subjects.

	Occupational mold exposure	Indoor (home) mold exposure
Number: n	23	13
Extensively exposed: n (%)	17 (74%)	6 (46%)
Currently exposed: n (%)	4 (17%)	5 (39%)
Low exposed: n (%)	2 (9%)	2 (15%)
Respiratory symptoms: n (%)	23 (100%)*	10 (77%)
Asthma: n (%)	20 (87%)**	6 (46%)
sx1 IgE ≥ 0.35 (kU_A_/L): n (%)	14 (61%)	10 (77%)
sx1 IgE (kU_A_/L): median (range)	0.65 (0.03 – 20.48)	15.62 (0.07 – 150.0)**
tIgE ≥ 150 (kU/L): n (%)	11 (48%)	8 (62%)
tIgE (kU/L): median (range)	140.8 (3.82 – 2122.0)	184.4 (18.15 – 4302.0)
mx1 IgE ≥ 0.35 (kU_A_/L): n (%)	9 (39%)	5 (39%)
mx1 IgE (kU_A_/L): median (range)	0.15 (0.02 – 26.31)	0.08 (0.02 – 16.63)
Re-testing of mx1 positive sera	n = 9	n = 5
Asp f sensitization: n (%)	9/9 (100%)	4/5 (80%)
Asp f IgE (kU_A_/L): median (range)	3.23 (0.93 – 61.63)*	0.69 (0.06 – 1.82)
Pen ch sensitization: n (%)	5/9 (56%)	4/5 (80%)
Pen ch IgE (kU_A_/L): median (range)	0.40 (0.07 – 8.7)	0.54 (0.04 – 6.21)
Alt a sensitization: n (%)	6/9 (67%)	5/5 (100%)
Alt a IgE (kU_A_/L): median (range)	1.12 (0.02 – 15.75)	1.83 (0.81 – 5.91)
Cla h sensitization: n (%)	3/9 (33%)	3/5 (60%)
Cla h IgE (kU_A_/L): median (range)	0.10 (0.02 – 2.02)	2.73 (0.03 – 18.42)
CC16 (ng/mL): median (range)	6.08 (3.99 – 21.96)	8.86 (5.29 – 16.16)
IL-6 (pg/mL): median (range)	4.7 (4.7 – 30.20)	4.7 (4.7 – 73.80)
SAA (ng/mL): median (range)	19,331 (1,880 – 95,488)	15,475 (3,086 – 578,522)

Significant differences are bold and labeled with *p ≤ 0.05 and **p ≤ 0.01.
